# Effect of Mo on the Mechanical and Corrosion Behaviors in Non-Equal Molar AlCrFeMnNi BCC High-Entropy Alloys

**DOI:** 10.3390/ma15030751

**Published:** 2022-01-19

**Authors:** Wei-Chen Hsu, Wei-Pin Kao, Jien-Wei Yeh, Che-Wei Tsai

**Affiliations:** 1 Department of Materials Science and Engineering, National Tsing Hua University, Hsinchu 30013, Taiwan; suprecrazy123123123@gmail.com (W.-C.H.); benkao20411295@gmail.com (W.-P.K.); jwyeh@mx.nthu.edu.tw (J.-W.Y.); 2 High Entropy Materials Center, National Tsing Hua University, Hsinchu 30013, Taiwan

**Keywords:** high-entropy alloys, body centered cubic, NiAl precipitates, high-temperature tensile, corrosion, EIS

## Abstract

Co-free body-centered cubic (bcc) high-entropy alloys (HEAs) are prepared, and the elevated mechanical property and corrosion property of the Al_0.4_CrFe_1.5_MnNi_0.5_Mo_x_ (x = 0 and 0.1) alloys are studied. The Vickers hardness (HV) of the as-homogenized state is between HV 350 and HV 400. Both alloys are provided with nano-scale NiAl-rich B2 precipitates which contribute to the strength at high-temperature. In addition, adding Mo in the present alloy strengthens by σ phase. Al_0.4_CrFe_1.5_MnNi_0.5_Mo_0.1_ exhibited outstanding tensile properties, with a yield strength of 413 MPa and ultimate tensile strength of 430 MPa in the elevated tensile test at 600 °C, which is better than that of Al_0.4_CrFe_1.5_MnNi_0.5_ alloy. Through potentiodynamic polarization testing in 0.5 M H_2_SO_4_ solution and electrochemical impedance spectroscopy (EIS), it is shown that adding Mo can effectively reduce the corrosion current density and improve the impedance of passive film, since the passivation layer is formed and stable.

## 1. Introduction

In 2004, a new alloy design concept called high-entropy alloys (HEAs) was proposed by Yeh et al. [1,2,3,4]. In recent years, it has also been used in various research topics, including ceramics [5,6], polymers [7], and composites [8]. Unlike traditional alloys that are primarily based on one or two elements. HEAs comprise five or more principal elements in equimolar or non-equimolar ratios, each at 5–35 at%. The four core effects of HEAs, namely high entropy, lattice distortion, sluggish diffusion, and cocktail effects, mean thaat HEAs possess numerous special properties that traditional alloys do not. Besides, the idea of mixing multiple elements while increasing configurational entropy (>1.5 R) to enhance high-temperature phase stability expands the potential of new alloy design. Along with several extraordinary properties, HEAs have been applied to various functions and applications [9,10,11,12,13]. Moreover, the corrosion resistance [14,15,16,17,18,19,20,21,22] and mechanical properties [23,24,25,26,27,28,29,30] in HEAs are widely investigated due to their importance and necessity, and these fields have huge potential for further development. Nowadays, most research focuses on single-phase FCC alloys, such as CoCrFeMnNi [31,32] and CoCrFeNi [33,34], as well as BCC refractory alloys. Since the first report of MoNbTaVW alloys, research on BCC refractory HEAs has blossomed, with over 20 different HEA systems [35,36,37,38,39,40,41,42]. Nevertheless, to the authors’ knowledge, the non-refractory BCC HEAs have been relatively overlooked from the mechanical point of view.

The Al_x_CrFe_1.5_MnNi_0.5_ (x = 0.3 and 0.5) non-equimolar alloy based on the Al_0.5_CoCrCuFeNi [43,44,45,46,47] was demonstrated to be composed of FCC and BCC phase when Al content was 0.3 mole, and BCC phase when Al content was 0.5 mole [48]. Both displayed significant high-temperature age hardening ability, which resulted from the formation of ρ phase (Cr_5_Fe_6_Mn_8_-like phase) [48], and an extensive corrosion passive region [49,50]. Moreover, cobalt-free alloy design can largely reduce the cost and broaden the applications. However, these alloys present low processability and low corrosion resistance. It is shown that the corrosion current density of Al_x_CrFe_1.5_MnNi_0.5_ is two orders of magnitude higher than 304 stainless steel [49]. Therefore, the improvement of alloy composition design is necessary.

Mo is considered a beneficial element to both solid solution strengthening and precipitation hardening [51]. Mo-containg HEAs generally exhibited high strength [52], good thermal stability [53], and corrosion resistance [54,55,56,57]. Niu et al. mentioned that Mo-oxide film induced by appropriate Mo addition can enhance the corrosion resistance [56]. Although the effect of adding Mo on corrosion and mechanical properties has been widely discussed in FCC HEAs [54,56], there are few studies on the effects of Mo addition for the BCC HEAs. On the other hand, the content of Al in the alloy will affect the corrosion properties as well. Lee et al. pointed out that increasing the content of Al in Al_x_CrFe_1.5_MnNi_0.5_ (x = 0, 0.3 and 0.5) would raise the corrosion current density and passive current density [49]. The characteristics of the elements mentioned above will be taken into consideration in alloy design.

In the present study, the Co-free BCC Al_0.4_CrFe_1.5_MnNi_0.5_Mo_x_ (x = 0 and 0.1) HEAs are investigated in order to maintain the single BCC matrix phase as well as enhance the corrosion resistance. The alloys will be prepared by reducing the Al content, and Mo addition can also improve the corrosion resistance [54,55,56,57]. With the examination of mechanical properties and corrosion resistance, a special focus is on the high-temperature tensile strength and corrosion current density. The electrochemical impedance spectroscopy (EIS) of these HEAs are also measured to observe the effect of Mo addition in the present alloys. In order to achieve industrial capacity, these properties would be a significant indicator.

## 2. Materials and Methods

The Al_0.4_CrFe_1.5_MnNi_0.5_Mo_x_ (x = 0 and 0.1) alloys were prepared for analysis by vacuum arc melting. The purity of every element was a higher level above 99.9 wt%. Ingots with a weight of approximately 100 g were re-melted at least three times to make sure the chemical composition uniformity of the alloys. The ingots were 75 mm in length, 25 mm in width, and 10 mm in thickness. All ingots were forged at 1200 °C with a 50% thickness reduction, then homogenized at 1200 °C for 6 h followed by air cooling.

The crystal structures of the alloys were analyzed by X-ray diffraction (XRD, Bruker D2 PHASER). The X-ray source was a Cu target and the diffractometer worked at 30 kV/10 mA with a characteristic wavelength λ (K_α1_) of 1.54056 Å. The angle of scanning ranged was from 20° to 100° and the scanning rate was 0.2°/s. The microstructures were obtained by scanning electron microscopy (SEM, JEOL JSM-IT100) operated at 20 kV. The chemical compositions were analyzed through energy dispersive X-ray spectroscopy (EDS). The SEM images of these alloys were taken without chemical etching. Thermo-Calc software was used to calculate the equilibrium phase as a function of temperature based on the CALPHAD method with the TCFE7 thermodynamic database. Tensile testing was performed with a universal testing system (SHIMADZU AGS-X 100 kN) equipped with a heating furnace (ATS series-3210) for temperature control. Flat dog-bone tensile specimens were electric discharge machined from the as-homogenized states with a gauge length of 19 mm, gauge width of 3.0 mm, and thickness of 1.5 mm. The engineering strain rate was 10^−3^ s^−1^ at elevated temperature. Each measurement was repeated three times for accuracy.

Both potentiodynamic polarization and electrochemical impedance spectroscopy (EIS) testing were conducted with a potentiostat (IVIUMnSTAT multichannel electrochemical analyzer) with a typical three electrode cell. An Hg/Hg_2_Cl_2_ electrode (saturated KCl) with E = −0.2412 V_SHE_ was used as the reference electrode and a platinum sheet was used as the counter electrode. The reacted surface area of 10 × 10 mm^2^ for all samples were ground to #1200 by SiC grit papers. The specimens were then tested in a 0.5 M H_2_SO_4_ solution at 25 °C under atmospheric pressure. The test solution was deaerated by bubbling purified nitrogen gas with the entire electrochemical tests to remove any effect of dissolved oxygen. In this case, it can be ensured that the electrochemical dissolution of the alloy is only contributed by the acidic solution (H_2_SO_4_). Potentiodynamic polarization curves and EIS were plotted after the specimen was allowed to corrode freely for 30 min, which is the time required to reach the quasi-steady-state value of open circuit potential (OCP). The potentiodynamic polarization tests were performed at a scan rate of 1 mV s^−1^ from an initial potential of −0.5 V to a final potential of 1.2 V versus to OCP. The EIS was conducted at OCP with a sinusoidal potential amplitude of 10 mV and an operating frequency of 100 kHz to 10 mHz. Following the potentiodynamic polarization and EIS testing, multiple electrochemical parameters were determined.

## 3. Results

### 3.1. Phase and Microstructure Analysis

**Figure 1:**
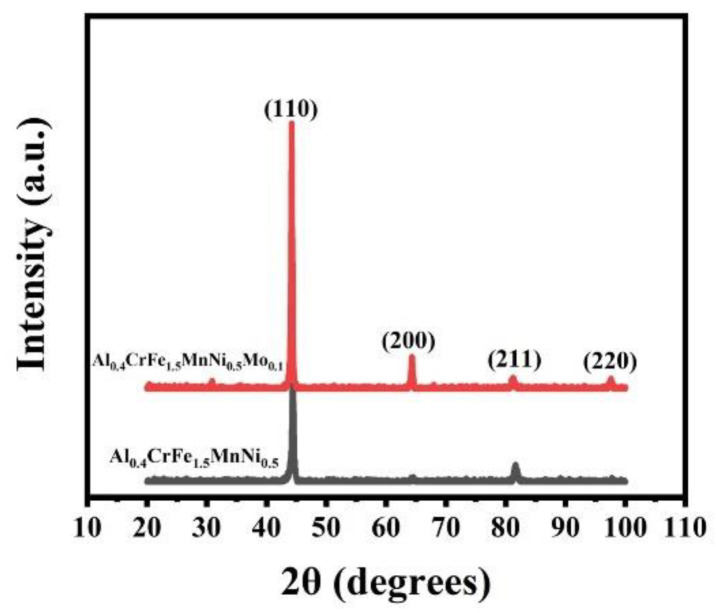
X-ray diffraction patterns of the alloys in as-homogenized state. Red: Al_0.4_CrFe_1.5_MnNi_0.5_Mo_0.1_; black: Al_0.4_CrFe_1.5_MnNi_0.5_.

Figure 1 shows the XRD patterns of the alloys in as-homogenized states. The crystal structures of the alloys all present a BCC solid solution structure. The lattice constants of Al_0.4_CrFe_1.5_MnNi_0.5_ and Al_0.4_CrFe_1.5_MnNi_0.5_Mo_0.1_ are 2.885 Å and 2.897 Å, respectively. This result is consistent with the Al_0.5_CrFe_1.5_MnNi_0.5_, which is a BCC solid solution structure in the previous study [48]. After adding Mo element, the increase in lattice constants is due to the contribution of Mo, whose radius is 141 p.m. [58], while the radii of the other elements except Al are approximately 125 p.m. [59]. Furthermore, it is noted that the (110) and (200) peak intensities of BCC phase in Al_0.4_CrFe_1.5_MnNi_0.5_Mo_0.1_ increase relative to Al_0.4_CrFe_1.5_MnNi_0.5_. This indicates the increase in the degree of (110) and (200) texture after adding Mo element.

**Figure 2:**
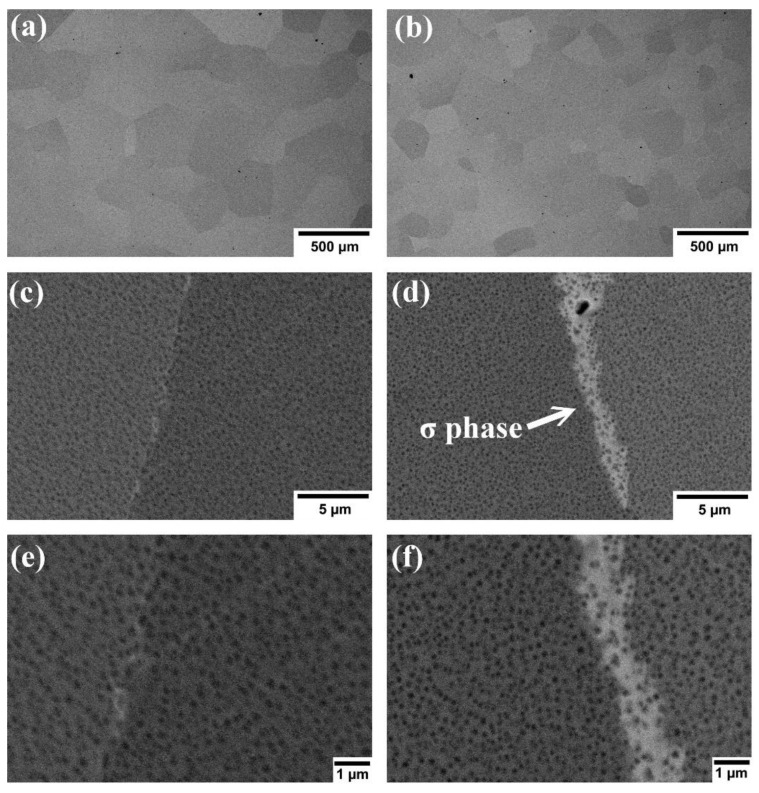
Microstructure of alloys: (**a**,**c**,**e**) are Al_0.4_CrFe_1.5_MnNi_0.5_, and (**b**,**d**,**f**) Al_0.4_CrFe_1.5_MnNi_0.5_Mo_0.1_ in backscatter electron images. Below is the high magnification surface of the specimens.

**Figure 3:**
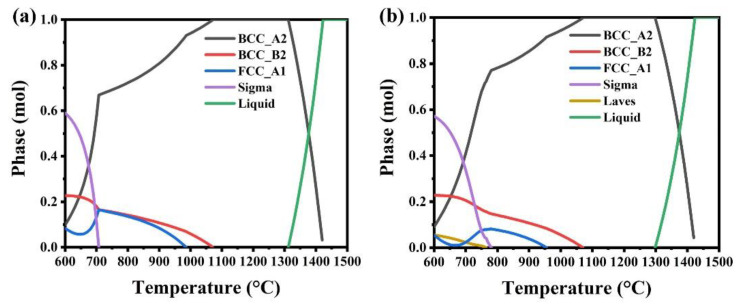
Thermo-Calc calculation diagrams, calculated using the CALPHAD method with the TCFE7 database, for (**a**) Al_0.4_CrFe_1.5_MnNi_0.5_ and (**b**) Al_0.4_CrFe_1.5_MnNi_0.5_Mo_0.1_.

As shown in Figure 2a,b with low magnification, the obvious grains can be observed. No dendritic structure is found in alloys. The grain sizes of Al_0.4_CrFe_1.5_MnNi_0.5_ and Al_0.4_CrFe_1.5_MnNi_0.5_Mo_0.1_ are 410 ± 101 μm and 284 ± 114 μm after 6 h homogenization, respectively. The grain size decreases from 410 μm to 284 μm are due to the addition of Mo. Since the melting temperature of Mo is 2896 K (2623 °C) and it is the highest one in this system, Mo can decrease the diffusion and form a refined crystal structure [60]. Figure 2c,d with high magnification indicate that both alloys have nano-scale precipitation in grains. According to previous studies [43,48], the fine gray precipitates in matrix are 7 nm to 50 nm and identified as the rich Al and Ni. In addition, Figure 3 shows that both alloys have a tendency of B2-type ordered phase formation before 1100 °C and single-phase BCC at 1200 °C. The B2 structure is an ordered bcc structure including two simple cubic interpenetrating sublattices. Among these B2-based intermetallic compounds, the most common compound is NiAl. A comparison with Figure 2c,d indicates that even though the two alloys underwent heat treatment at 1200 °C, they are still not single-phase. It can be concluded that the precipitates are thus a solution phase of the NiAl compound with ordered BCC (B2-type), where there were elemental replacement. In other words, Fe and Mn for partial Ni, and Cr for partial Al. From a high magnification microstructure, the precipitate size of Figure 2e is 43 nm, and the size of Figure 2f is 26 nm. It can be inferred that adding Mo also reduced the particle size, which may be due to the slow grain growth of the samples.

The microstructure in Figure 2d shows white phase on the grain boundary, indicating that Al_0.4_CrFe_1.5_MnNi_0.5_Mo_0.1_ alloy has composition segregation on the grain boundary. According to Table 1, the white region along grain boundaries in Al_0.4_CrFe_1.5_MnNi_0.5_Mo_0.1_ alloy has higher Cr and Mo contents and a lower Ni content than the gray phase region, which is known to be the σ phase [61,62]. The σ phase is mostly found in the iron-chromium-molybdenum system. Its crystal structure is defined as the topologically close-packed (TCP) phase, which is observed in many intermetallic compounds. However, it can be found in Figure 3b that the σ phase cannot form above 800 °C. This contradiction is owing to hot forged behavior which was led mainly by dynamic recrystallization [63]. There are more heterogeneous nucleation sites such as grain boundaries, so the complex σ phase is difficult to completely melt back into the matrix. Moreover, the σ phase formation tendency of austenitic stainless steel can be known from the equivalent chromium content (ECC) equation in weight percent [64]:

ECC = % chromium (Cr) + 0.31% manganese (Mn) + 1.76% molybdenum (Mo) + 0.97% tungsten (W) + 2.02% vanadium (V) + 1.58% silicon (Si) + 2.44% titanium (Ti) + 1.7% niobium (Nb) + 1.22% tantalum (Ta) − 0.226% nickel (Ni) − 0.177% cobalt (Co)(1)

If the ECC is greater than 17–18%, the steel tends to form σ phase. In this case, the ECC value for the Al_0.4_CrFe_1.5_MnNi_0.5_Mo_0.1_ alloy was 32.8%. This indicates that the formation of σ phase in the Mo-containing alloy is reasonable.

### 3.2. High-Temperature Tensile Results

**Figure 4:**
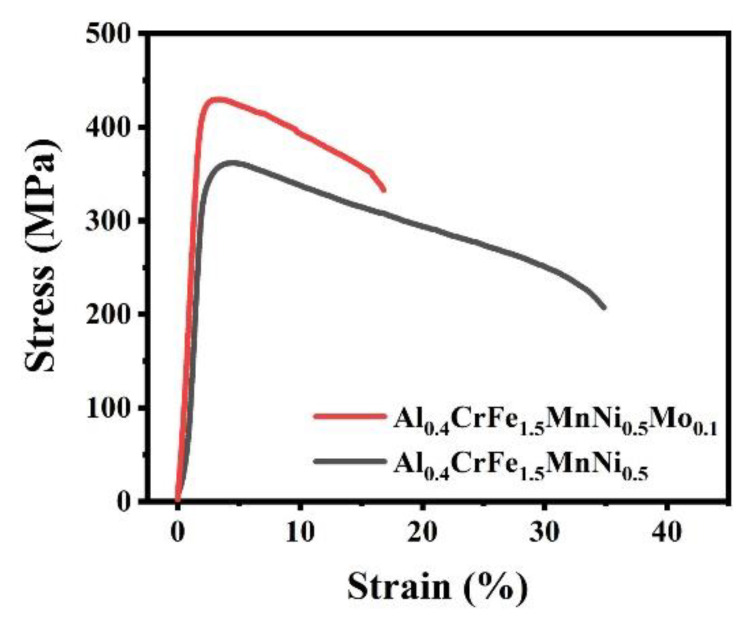
Tensile engineering stress-strain curve of Al_0.4_CrFe_1.5_MnNi_0.5_ and Al_0.4_CrFe_1.5_MnNi_0.5_Mo_0.1_ alloys at 600 °C.

Since both the alloys demonstrate excellent hardening ability, the mechanical properties of the Al_0.4_CrFe_1.5_MnNi_0.5_ and the Al_0.4_CrFe_1.5_MnNi_0.5_Mo_0.1_ at high-temperature were examined. Figure 4 shows the engineering tensile stress-strain curves at 600 °C. The yield strength of the Al_0.4_CrFe_1.5_MnNi_0.5_ is above 300 MPa and the Al_0.4_CrFe_1.5_MnNi_0.5_Mo_0.1_ exceeds 400 MPa even at 600 °C. The yield strength (YS), ultimate tensile strength (UTS), and elongation (EL) of the alloys are listed in Table 2. The volume fraction of the B2-type NiAl precipitate is higher than 30%, and it can sustain high strength at high-temperature. Meanwhile, the Al_0.4_CrFe_1.5_MnNi_0.5_Mo_0.1_ shows higher yield strength than the Al_0.4_CrFe_1.5_MnNi_0.5_, and this is due to the precipitation hardening by σ phase. Moreover, both the alloys demonstrate the best ductility at 600 °C, especially Al_0.4_CrFe_1.5_MnNi_0.5_, at up to 32%. These results indicate that the Al_0.4_CrFe_1.5_MnNi_0.5_ and the Al_0.4_CrFe_1.5_MnNi_0.5_Mo_0.1_ show a good strength–ductility balance at elevated temperature. The mechanisms of high-temperature strength and ductility will be discussed in detail later.

**Figure 5:**
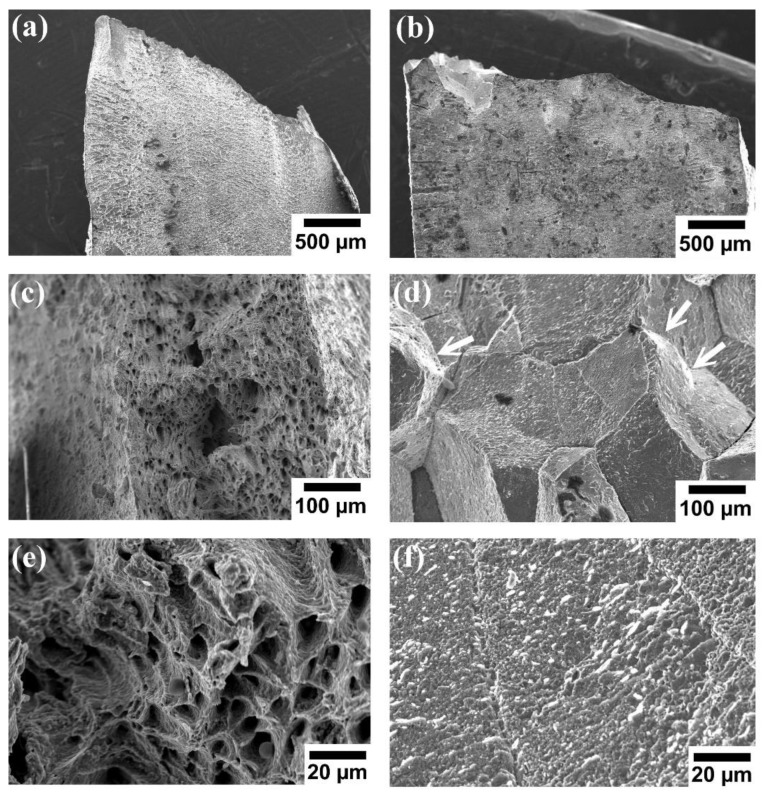
Fractography of Al_0.4_CrFe_1.5_MnNi_0.5_Mo_x_ alloys: (**a**,**c**,**e**) x = 0 and (**b**,**d**,**f**) x = 0.1. Below is the high magnification surface of the specimens. The white arrows are the positions of the microridge structures.

Figure 5 shows the fracture surface of both alloys after tensile tests at 600 °C. The fracture surface of Al_0.4_CrFe_1.5_MnNi_0.5_ alloy shows a 45 degree fracture, indicating that a ductile rupture was formed, as shown in Figure 5a. In addition, the fracture surface under micro view exhibited many ductile dimples, as shown in Figure 5c,e. This further confirms that Al_0.4_CrFe_1.5_MnNi_0.5_ alloy is a typical ductile fracture. On the other hand, Figure 5b illustrates the flat fracture surface of Al_0.4_CrFe_1.5_MnNi_0.5_Mo_0.1_ alloy. The obvious intergranular fracture owing to the brittle σ phase precipitation along grain boundaries can be found, as shown in Figure 5d. Besides, the typical brittle fracture pattern is directly related to the brittle σ phase precipitated in Al_0.4_CrFe_1.5_MnNi_0.5_Mo_0.1_ alloy, as shown in Figure 5f. However, Figure 5d shows the microridge structures (white arrow) formed on the grains, revealing local ductility in the alloy. So, Al_0.4_CrFe_1.5_MnNi_0.5_Mo_0.1_ alloy is still provided with a slightly ductile instead of completely brittle fracture.

### 3.3. Electrochemical Properties Analysis

**Figure 6:**
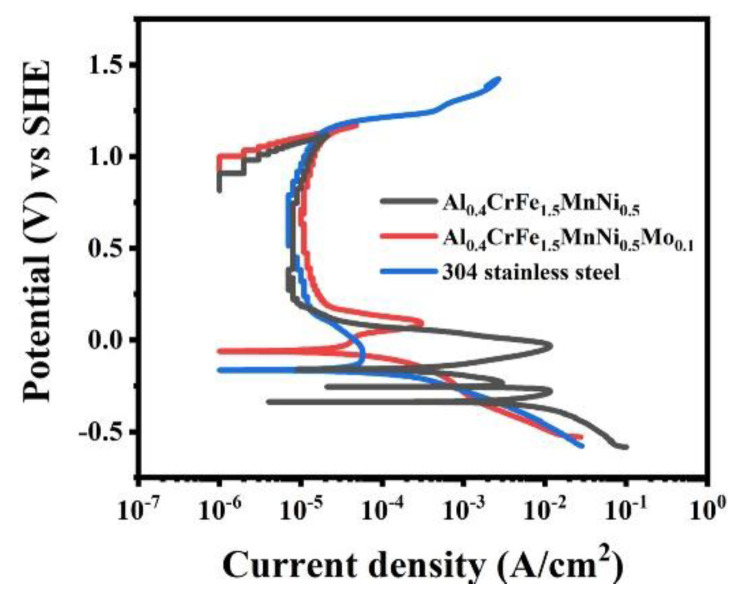
Potentiodynamic polarization curves of the alloys in 0.5 M H_2_SO_4_ solution.

Figure 6 plots the potentiodynamic polarization curves of the alloys in 0.5 M H_2_SO_4_ solution. All alloys present a typical passive region. Table 3 summarizes the values of electrochemical parameters for the alloys in 0.5 M H_2_SO_4_ solution. The corrosion current densities (I_corr_) of the Al0.4CrFe1.5MnNi0.5Mo_x_ alloy are determined to fall from 3200 μA/cm^2^ to 61 μA/cm^2^ as the Mo content increased to 0.1 mol. The corrosion potentials (E_corr_) of Al_0.4_CrFe_1.5_MnNi_0.5_ and Al_0.4_CrFe_1.5_MnNi_0.5_Mo_0.1_ are −340 and −60 mV, respectively. Based on the above results, the addition of 0.1 mol Mo can inhibit the reduction reaction of hydrogen ions in the solution and greatly increase the corrosion resistance. Furthermore, the critical current density (I_crit_) would drastically drop from 1.1 × 10^−2^ A/cm^2^ to 2.7 × 10^−4^ A/cm^2^ when the alloy is supplemented with Mo, implying that Al_0.4_CrFe_1.5_MnNi_0.5_ faces a greater obstacle before entering passivation. Critical pitting potential (Epit) is the minimum positive potential at which the metal salt of the aggressive ion in solution is in equilibrium with the metal oxide. All alloys present similar values of Epit. This is due to the continuous increase of applied potential, and the release of oxygen after reaching the electrolysis potential of water (1.23V) becomes the main reaction mechanism. In addition, the potentiodynamic polarization curve shows that the Al_0.4_CrFe_1.5_MnNi_0.5_Mo_0.1_ has a negative and large hysteresis loop, which means that the metal is still well protected after passivation.

**Figure 7:**
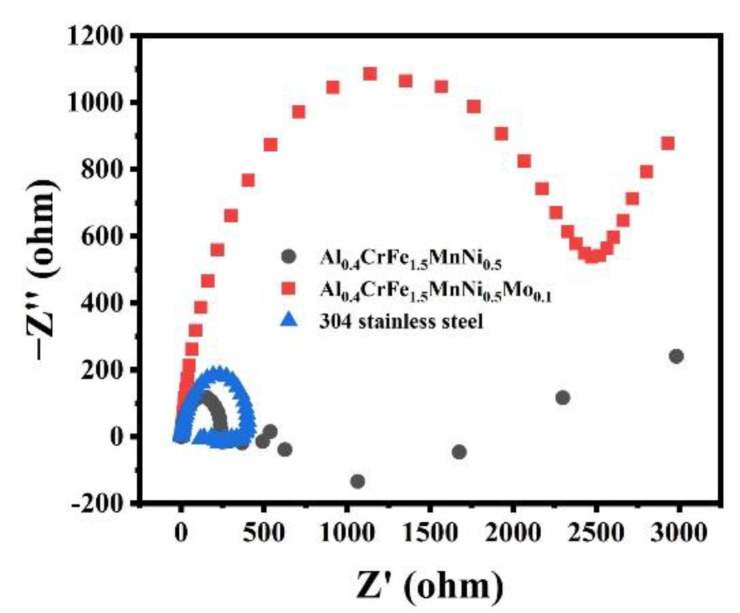
Nyquist plot of the alloys in 0.5 M H_2_SO_4_ solution.

To further investigate the interface of the corrosion reaction between solution and alloys, the electrochemical impedance spectroscopy test of the alloys in 0.5 M H_2_SO_4_ solution are shown in Figure 7. The Nyquist plot of the Al_0.4_CrFe_1.5_MnNi_0.5_Mo_0.1_ and 304 stainless steel alloys include one capacitive loop from high to low frequencies, revealing that the alloys are provided with a passivation layer when corroded. Al_0.4_CrFe_1.5_MnNi_0.5_Mo_0.1_ alloys only include one capacitive loop, resulting from the presence of Mo element in HEA. This element is present in the outer regions of the passive film as Mo(VI) and generates in its unoxidized state at the metallic matrix immediately beneath the film, which can be used as a barrier and inhibit the formation of adsorption layer [57]. On the other hand, a different behavior was observed in Al_0.4_CrFe_1.5_MnNi_0.5_ alloy. It includes two capacitive loops in electrochemical impedance diagrams, which are typically related to the presence of a passivation layer and an adsorption layer. The reason for the formation of the adsorption layer is that the elements, such as Al, Cr, and Ni, in the alloy dissolve in the solution, forming compounds with hydroxide or sulfate ions and adsorbing on the metal surface, thus forming the adsorption layer [65,66].

**Figure 8:**
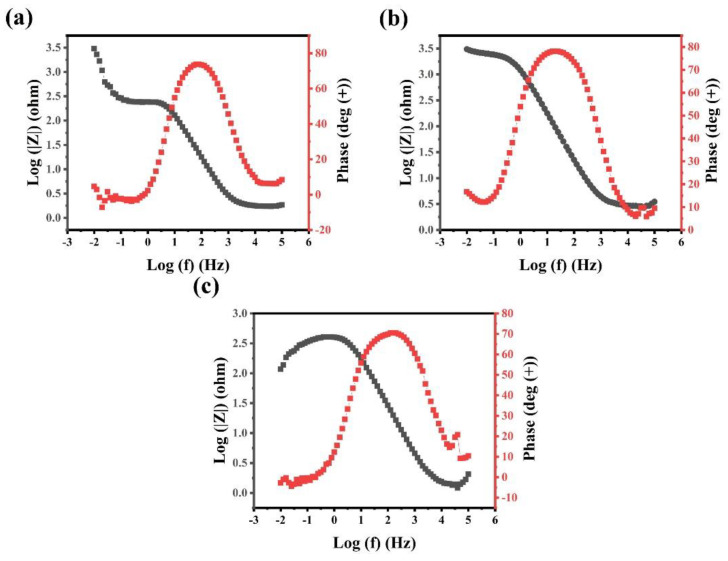
Bode plot of (**a**) Al_0.4_CrFe_1.5_MnNi_0.5_ (**b**) Al_0.4_CrFe_1.5_MnNi_0.5_Mo_0.1_ (**c**) 304 stainless steel in 0.5 M H_2_SO_4_ solution.

Figure 8 presents the Bode plots of the alloys. Similar to the previous results, the phase in Figure 8a has two peaks and the impedance changes significantly at low frequencies, indicating that two layers are produced in Al_0.4_CrFe_1.5_MnNi_0.5_ alloy. The other two alloys have one phase peak as shown in Figure 8b,c, which means that only one passivation layer is formed. Moreover, Table 4 summarizes the simulated values for the equivalent circuit elements. R_s_ is the resistance of the solution; R_pass_ is the charge transfer resistance that is associated with the protection ability of passivation layer; R_ad_ represents the resistance of the adsorptive film on the metal surface, while Q_pass_ and Q_ad_ are the capacitance of passive layer constant phase element and the capacitance of adsorptive film constant phase element, respectively. Based on above results, the impedance value of the passive layer (R_pass_) of Al_0.4_CrFe_1.5_MnNi_0.5_Mo_0.1_ is much greater than Al_0.4_CrFe_1.5_MnNi_0.5_ and 304 stainless steel during passivation, showing that the passivation layer of Al_0.4_CrFe_1.5_MnNi_0.5_Mo_0.1_ possesses better protection ability. To sum up, the corrosion current density and corrosion potential of Al_0.4_CrFe_1.5_MnNi_0.5_Mo_0.1_ in 0.5 M H_2_SO_4_ are better than those of Al_0.4_CrFe_1.5_MnNi_0.5_ and 304 stainless steel alloys. Furthermore, the impedance of the Al_0.4_CrFe_1.5_MnNi_0.5_Mo_0.1_ passivation layer is the largest, indicating that its corrosion resistance is the best in three alloys. 

## 4. Discussion

### 4.1. Elongation Behavior of the Alloys at High-Temperature

Both alloys cracked within 5% of rolling at room temperature and showed the characteristics of a complete brittle fracture (not shown here). However, as shown in Figure 5c,e, the ductile fracture mode, such as dimples and microvoids, can be seen on the fracture surface of the alloys. Moreover, their elongation is greatly increased at 600 °C, especially Al_0.4_CrFe_1.5_MnNi_0.5_ alloy. The reason for the increase in elongation is that the alloys experience brittle to ductile transition at high-temperature. In BCC HEAs, the Peierls–Nabarro stress is expected to be very high because the slip plane consists of different atoms with different sizes, which may increase the lattice resistance to the movement of dislocations [67].

The nucleation and propagation of double kink is a mechanism to overcome lattice resistance through the motion of a/2 <111> screw dislocation in bcc metals [68]. Kink will ease the movement of dislocations through the lattice. Since the kinked sections are located across higher energy portions of the crystal, they can slip more simply than the line segment along the energy troughs, which must overcome the largest energy barrier if they move. However, kink formation is a thermally activated process [69], which implies that at high-temperature the number of double kinks is just enough to let dislocation slip [70]. Hence, the elongation of the both alloys is increased substantially at high-temperature.

### 4.2. Effects of the NiAl Precipitates and σ Phase on Deformation Behavior at High-Temperature

According to a previous study [48], the Al_x_CrFe_1.5_MnNi_0.5_ (x = 0.3 and 0.5) series alloy exhibits a large age hardening effect at temperatures from 600 to 800 °C. For instance, the hardness of both alloys increases from Hv 300 to Hv 550 in 1 h. The aging hardening ability is due to the formation of ρ phase (Cr_5_Fe_6_Mn_8_-like phase), which displays a very high hardness of Hv 1273. However, the hardness of the alloys in this study falls between 350 and 400 HV whether the alloys have undergone tensile testing at 600 °C. It can be inferred that the ρ phase is not formed during tensile testing, and there is no strengthening contribution of the ρ phase.

On the other hand, as shown in Figure 2b,d, both alloys disperse with nano B2-type NiAl precipitates in the matrix. Fine NiAl precipitates with diameters of 10–30 nm were found to significantly strengthen the Fe-Al-Ni-based alloy, which is because of the difference primary slip system between the BCC matrix and the B2 precipitates [71]. The nano-scale NiAl precipitates can also be found in HEAs resulting in improved mechanical properties [72,73]. Generally, the {101} <111> slip occurs in BCC metals while B2 crystals are known to deform by {110} <001> slip at room temperature [74]. The activation of <111> slip unfavorable for the NiAl precipitates leads to great hardening ability during tensile testing, called slip frustration hardening (SFH) [75]. However, the slip system changes from <111> to <001> at 650 °C because the critical resolved shear stress for <001> slip of the B2 crystals drop rapidly with increasing temperature [76]. This means that the NiAl precipitates are sheared by <001> dislocations and bypassed by the 1/2 <111> dislocations, resulting in the strengthening of alloys by both SFH and the Orowan strengthening at elevated temperature [75]. As a result, alloys containing B2-type NiAl precipitates and BCC matrix exhibit excellent elevated mechanical strength. Further experiments are required to confirm the formation of the slip system and dislocation movement in Al_0.4_CrFe_1.5_MnNi_0.5_Mo_x_ (x = 0 and 0.1) alloys.

Al_0.4_CrFe_1.5_MnNi_0.5_Mo_0.1_ had a hardness of about 30HV lower than Al_0.4_CrFe_1.5_MnNi_0.5_, but it exhibited better yield strength (YS) and ultimate tensile strength (UTS) at high-temperature. Since the σ phase was harder, it could be used as an obstacle to dislocation when stretched. Wang et al. [77] stated that the harder σ phase could hinder the dislocation movement during the tensile process and finally enhance the strength of duplex stainless steel, but the elongation would decrease. Zhang et al. [78] also reported that the low volume fraction of the nano-σ particles in FCC HEAs can strengthen the alloy with less sacrifice of ductility. Therefore, the Al_0.4_CrFe_1.5_MnNi_0.5_Mo_0.1_ alloy possesses better mechanical strength without sacrificing lots of ductility.

### 4.3. Effects of Mo Addition on the Corrosion Behavior

The beneficial effects of adding Mo are widely recognized with regard to the corrosion resistance of stainless steel [79,80,81]. Sugimoto and Sawada [82] stated that the addition of Mo to austenitic stainless steel can form a passive film composed of a solid solution of Mo^6+^ in a chromium oxyhydroxide network, which drops the corrosion current density in the active region for acidic solution. However, Hashimoto et al. [83] proposed that the main role of Mo is to reduce the dissolution rate of the active zones by forming and retaining Mo (VI) oxyhydroxide or molybdate (MoO_4_^2^^−^) at these positions.

As mentioned above, many authors conclude that molybdenum in the passivation layer is primarily an ionic compound (MoO_4_^2^^−^) that adsorbs on top of the material surface and acts as a barrier to electrochemical attack [81,84,85]. However, given the strong acidic conditions (pH < 1) and the corrosion potential of the materials in the present studied solution, the influence of Mo through molybdate formation should be considered impossible according to the Pourbaix diagrams [86]. Since molybdate is thermodynamically unstable, it tends to precipitate as MoO_3_ oxide under these conditions. MoO_3_ is a protective oxide layer, which can decrease the rate of the corrosion process, leading to an improvement in the corrosion behavior in H_2_SO_4_ solution. Hence, Al_0.4_CrFe_1.5_MnNi_0.5_Mo_0.1_ alloy possesses better corrosion resistance than Al_0.4_CrFe_1.5_MnNi_0.5_ alloy owing to the MoO_3_ protective oxide layer.

## 5. Conclusions

Adding Mo to the Al_0.4_CrFe_1.5_MnNi_0.5_Mo_x_ (x = 0 and 0.1) alloy strengthens with σ phase. The body-centered cubic (bcc) high-entropy alloys (HEAs) improved the elevated mechanical property and corrosion property at the same time.

1. The present alloys are all BCC solid-solution structures and disperse with the nano B2-type NiAl precipitates in the matrix. Moreover, Al_0.4_CrFe_1.5_MnNi_0.5_Mo_0.1_ exists as σ phase with rich Cr and Mo contents and a lack of Ni content along the grain boundary.
2. The mechanical properties of the alloys improve upon Mo addition, reaching a yield strength of 413 MPa, an ultimate tensile strength of 430 MPa, and an elongation of 14.7% at 600 °C. This is ascribed to the slip frustration hardening (SFH) of nano NiAl precipitates and the precipitation hardening of σ phase. The results indicate that Al_0.4_CrFe_1.5_MnNi_0.5_Mo_0.1_ shows good strength–ductility balance at elevated temperature.
3. Al_0.4_CrFe_1.5_MnNi_0.5_Mo_0.1_ alloy exhibits superior corrosion resistance compared to Al_0.4_CrFe_1.5_MnNi_0.5_ and 304 stainless steel. In addition, the impedance of the Al_0.4_CrFe_1.5_MnNi_0.5_Mo_0.1_ passivation layer is the largest, indicating that its passive film is formed and stable. The results agree with the formation of the MoO_3_ protective layer. Therefore, we can conclude that Mo addition improves the corrosion resistance of HEAs.

## Figures and Tables

**Table 1:** Chemical compositions from EDS analysis of the alloys (unit: at.%).

Alloys	Elements	Al	Cr	Fe	Mn	Ni	Mo
Al_0.4_CrFe_1.5_MnNi_0.5_	matrix	9.1 ± 0.6	23.7 ± 0.2	32.2 ± 0.6	23.8 ± 0.4	11.1 ± 0.1	-
Al_0.4_CrFe_1.5_MnNi_0.5_Mo_0.1_	matrix	8.4 ± 0.5	22.5 ± 0.5	31.7 ± 0.4	24.9 ± 0.4	10.2 ± 0.2	2.3 ± 0.2
	Gray	8.9 ± 0.3	23.6 ± 0.5	34.7 ± 0.2	20.8 ± 0.4	9.4 ± 0.3	2.6 ± 0.2
	White	7.8 ± 0.4	24.8 ± 0.3	34.3 ± 0.1	22.2 ± 0.2	7.76 ± 0.4	3.15 ± 0.1

**Table 2:** The YS, UTS, and EL of the alloys at 600 °C.

Alloys	YS (MPa)	UTS (MPa)	EL (%)
Al_0.4_CrFe_1.5_MnNi_0.5_	307	361	32.7
Al_0.4_CrFe_1.5_MnNi_0.5_Mo_0.1_	413	430	14.7

**Table 3:** E_corr_, I_corr_, I_pass_, and E_pit_ of the alloys in 0.5 M H_2_SO_4_ solution.

Alloys	I_corr_ (μA/cm^2^)	E_corr_ (mV_SHE_)	I_crit_ (μA/cm^2^)	I_pass_ (μA/cm^2^)	E_pit_ (mV_SHE_)
Al_0.4_CrFe_1.5_MnNi_0.5_	3200	−340	11,000	8	1120
Al_0.4_CrFe_1.5_MnNi_0.5_Mo_0.1_	61	−60	270	10	1120
304 stainless steel	72	−160	58	7	1150

**Table 4:** Equivalent circuit elements values for EIS data corresponding to the alloys in 0.5 M H_2_SO_4_ solution.

Alloys	R_s_ (ohm)	R_pass_ (ohm)	Q_pass_ (μS-s^n^/cm^2^)	R_ad_ (ohm)	Q_ad_ (μS-s^n^/cm^2^)
Al_0.4_CrFe_1.5_MnNi_0.5_	1.8	30.8	72.9	233.9	33.4
Al_0.4_CrFe_1.5_MnNi_0.5_Mo_0.1_	2.9	2348	127	-	-
304 stainless steel	1.4	252.7	115.5	-	-

## Data Availability

The data presented in this study are available on request from the corresponding author and data is contained within the article.
